# Fertility-Sparing Treatment for Early-Stage Cervical Cancer ≥ 2 cm: A Problem with a Thousand Nuances—A Systematic Review of Oncological Outcomes

**DOI:** 10.1245/s10434-022-12436-w

**Published:** 2022-09-05

**Authors:** Carlo Ronsini, Maria Cristina Solazzo, Nicolò Bizzarri, Domenico Ambrosio, Marco La Verde, Marco Torella, Raffaela Maria Carotenuto, Luigi Cobellis, Nicola Colacurci, Pasquale De Franciscis

**Affiliations:** 1 Department of Woman, Child and General and Specialized Surgery, University of Campania “Luigi Vanvitelli”, Naples, Italy; 2 Unit of Gynecologic Oncology, Department of Woman, Child and Public Health, A. Gemelli, IRCCS, University Hospital Foundation, Rome, Italy

## Abstract

**Background:**

Fertility-sparing treatments (FSTs) have played a crucial role in the management of early-stage cervical cancer (ECC). The guidelines have recognized various approaches, depending on the tumor stage and other risk factors such as histotype and lymphovascular positivity. Much more debate has centered around the boundary within which these treatments should be considered. Indeed, these are methods to be reserved for ECC, but tumor size may represent the most significant limitation. In particular, there is no consensus on the strategy to be adopted in the case of ECC ≥ 2 cm. Therefore, this systematic review was to collect the literature evidence regarding the management of these patients.

**Methods:**

Following the recommendations in the Preferred Reporting Items for Systematic Reviews and Meta-Analyses (PRISMA) statement, we systematically searched the Pubmed and Scopus databases was conducted in April 2022, from the date of the first publication. We made no limitation on the country. We included all studies containing data on disease-free survival, overall survival, recurrence rate (RR), or complete response rate (CRR) to chemotherapy.

**Results:**

Twenty-six studies fulfilled the inclusion criteria, and 691 patients were analyzed regarding FST. Surgery-based FST showed an RR of between 0 and 42.9%, which drops to 12.9% after excluding the vaginal or minimally invasive approaches. Furthermore, papers regarding FST based on the neoadjuvant chemotherapy (NACT) approach showed a CRR of between 21.4 and 84.5%, and an RR of between 0 and 22.2%

**Conclusion:**

This paper focused on the significant heterogeneity present in the clinical management of FST of ECC ≥ 2 cm. Nevertheless, from an oncological point of view, approaches limited to the minimally invasive or vaginal techniques showed the highest RR. Vice versa, the lack of standardization of NACT schemes and the wealth of confounders to be attributed to the histological features of the tumor make it difficult, if not impossible, to set a standard of treatment.

**Supplementary Information:**

The online version contains supplementary material available at 10.1245/s10434-022-12436-w.

Although the incidence of cervical cancer has shown a downward trend in recent decades, in Western countries the age of first pregnancy has shown an opposite trend, raising its threshold.^1,2^ This has resulted in a possible overlapping, making it increasingly common for patients to be diagnosed with early-stage cervical carcinoma (ECC) who have not yet completed their reproductive expectations. Therefore, fertility-sparing treatments (FSTs) have played a crucial role in patient management. The guidelines recognize various approaches,^3,4^ depending on the tumor stage and other risk factors such as histotype and lymphovascular positivity.^5^ Much more debate has centered around the boundary within which these treatments should be considered. Indeed, these are methods are to be reserved for ECC, but tumor size may represent the most significant limitation. In particular, there is no consensus on the strategy to be adopted in the case of ECC ≥ 2 cm.^5^ In fact, of the five possible principal FSTs (conization, vaginal trachelectomy, minimally invasive trachelectomy, abdominal trachelectomy, and neoadjuvant chemotherapy combined with conization or trachelectomy), there is evidence of the inadequacy of the vaginal trachelectomy approach described by Dargent.^6,7^ The minimally invasive and abdominal approaches should also be set aside in parallel with the valid evidence for traditional radical hysterectomy surgery.^8^ Conversely, there is no consensus on the gold standard between radical abdominal approaches and neoadjuvant chemotherapy (NACT). Many referral centers use opposing strategies, making this field an open debate. In addition, more confusion is related to the fact that tumors ≥ 2 cm in size are at higher risk of recurrence regardless of FST. FST should be considered a non-routine approach for the management of ECC ≥ 2 cm and deserves further investigation. Therefore, this systematic review aims to collect all the literature evidence regarding the management of these patients.

## Material and Methods

The methods for this study were specified *a priori* based on the recommendations in the Preferred Reporting Items for Systematic Reviews and Meta-Analyses (PRISMA) statement.^9^ We registered the review in the PROSPERO database for meta-analysis (protocol number CRD42022316650).

### Search Method

We conducted a systematic search of the Pubmed and Scopus databases in April 2022, from the date of the first publication, for articles on oncological outcomes in FST of ECC ≥ 2 cm. The search was restricted to studies published in the English language, and no restrictions on country were made. Search imputes were ‘fertility sparing’ and ‘cervical neoplasm’.

### Study Selection

**Fig. 1.**
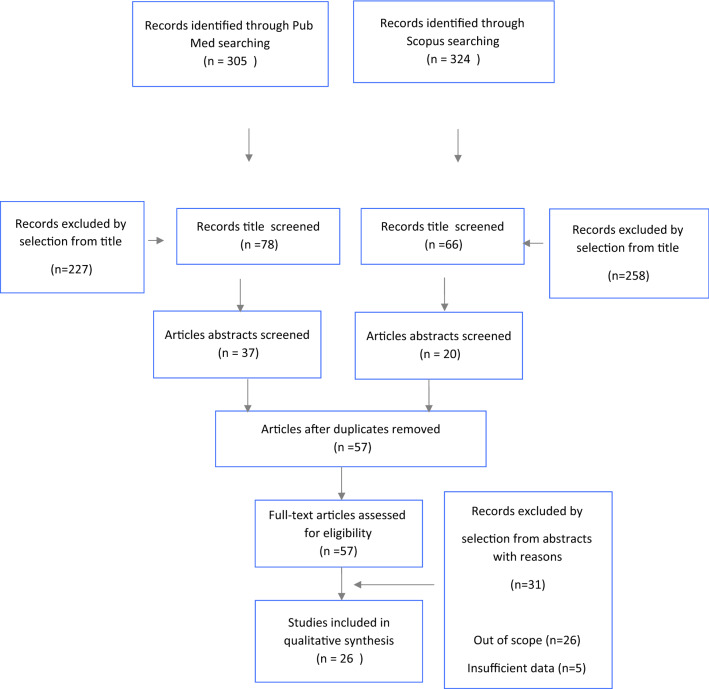
PRISMA flow diagram

Study selection was made independently by MCS and RC, and in the case of discrepancies, CRR decided on the inclusion/exclusion of the studies. Inclusion criteria were (1) studies that included patients with ECC > 2 cm; and (2) studies that reported at least one outcome of interest (overall survival [OS], disease-free survival [DFS], recurrence rate [RR], and complete response rate [CRR]). We excluded peer-reviewed articles, either if published originally, as well as non-original studies, preclinical trials, animal trials, abstract-only publications, and articles in a language other than English. If possible, the authors of studies that were only published as congress abstracts were contacted via email and asked to provide their data. The Preferred Reporting Items for Systematic Reviews and Meta‐Analyses (PRISMA) flowchart (Fig. 1) details the studies selected and the reasons for exclusion. All included studies were assessed regarding potential conflicts of interest.

### Data Extraction

MCS and RC extracted the data for all relevant series and case reports, as well as data on tumor characteristics (size, stage, histological subtype, lymphovascular space invasion [LVSI] status, grading), surgical approach, morbidity, and oncological issues such as recurrences, deaths, RR, and CRR to chemotherapy treatment (CR). Where possible, data on positive or close margins (taken to be indicative of inadequate surgical treatment), deep stromal infiltration (expressed as a percentage of patients with stromal infiltration ≥ 50%), and the distance between the tumor and internal uterus orefix were extracted; however, this activity was hindered by different criteria across papers and a diffused lack of information.

### Quality Assessment

We assessed the quality of the included studies using the Newcastle–Ottawa scale (NOS).^10^ This assessment scale uses three broad factors (selection, comparability, and exposure), with the scores ranging from 0 (lowest quality) to 8 (best quality). Two authors (MT and MLV) independently rated the quality of the studies and any disagreement was subsequently resolved by discussion or consultation with a third author (CR). The NOS is reported in the electronic supplementary material.

## Results

### Study Characteristics

After the database search, 629 articles matched the search criteria. After removing records with no full text, duplicates, and articles with the wrong study design (e.g., reviews), 32 studies remained that met the eligibility criteria, of which 26 matched the inclusion criteria and were included in the systematic review. All of these studies were retrospective or prospective studies evaluating surgical approaches such as abdominal radical trachelectomy (ART), laparoscopic radical trachelectomy (LRT), robotic radical trachelectomy (RRT), and vaginal radical trachelectomy (VRT), or NACT followed by minor surgery (conization or vaginal trachelectomy) or the other technique mentioned (Fig. 1). A few of these studies reported a comparison between two or more surgical approaches, and none of them reported a direct comparison between ART and NACT. Table 1 summarizes the country where the study was conducted, publication year, study design, International Federation of Gynecology and Obstetrics (FIGO) tumor stage, and number of participants.^11–36^

**Table 1:** Study characteristics

References	Country	Study design	Study year	FIGO stage	No. of participants
Cao et al.^11^	China	Prospective, case-control, multicentric study	2003–2012	IB1 > 2 cm^a^	48
De Vincenzo et al.^12^	Italy	Retrospective, observational, monocentric study	2014–2018	IB2	13
Deng et al.^13^	China	Retrospective, observational, monocentric study	–	IB1 > 2 cm^a^	45
Guo et al.^14^	China	Retrospective, observational, monocentric study	2003–2016	IB1 > 2 cm^a^	71
Kim et al.^15^	Korea	Retrospective, observational, monocentric study	2004–2009	IB1 > 2 cm^a^	32
Lanowska et al.^16^	Germany	Retrospective, observational, monocentric study	2006–2013	IB1 > 2 cm^a^ IB2	18
Li et al.^17^	China	Retrospective, observational, monocentric study	2004–2010	IB1 > 2 cm^a^	14
Li et al.^18^	China	Retrospective, observational, monocentric study	2004–2017	IB1 > 2 cm^a^	132
Lintner et al.^19^	Hungary UK USA	Retrospective, observational, multicentric study	1999–2006	IB1 > 2 cm^a^ IB2	31
Lu et al.^20^	China	Retrospective, observational, monocentric study	2005–2012	IB1 > 2 cm^a^	6
Marchiole et al.^21^	France	Retrospective, observational, monocentric study	2007–2010	IB–IIA > 2 cm^a^	7
Marchiole et al.^22^	France	Retrospective, observational, monocentric study	2007–2017	IB1 > 2 cm^a^ IB2 IIA1 > 2 cm^a^	19
Okugawa et al.^23^	Japan	Retrospective, observational, monocentric study	2001–2011	IB1 > 2 cm^a^	77
Pahisa et al.^24^	Spain	Retrospective, observational, monocentric study	1996–2007	IB1 > 2 cm^a^	4
Park et al.^25^	Korea	Retrospective, observational, multicentric study	2004–2012	IB1 > 2 cm^a^ IB2^a^	29
Plante et al.^26^	Canada	Prospective, observational, monocentric study	1991–2010	IB1 > 2 cm^a^ IB2^a^	14
Rendón et al.^27^	Colombia	Retrospective, observational, monocentric study	2009–2019	IB1 > 2 cm^a^ IB2^a^ IIA1 > 2 cm^a^	25
Robova et al.^28^	Czech Republic	Retrospective, observational, monocentric study	2005–2013	IB1 > 2 cm^a^ IB2^a^	28
Salihi et al.^29^	Belgium	Retrospective, observational, monocentric study	2004–2013	IB1 > 2 cm^a^ IB2^a^	5
Slama et al.^30^	Czech Republic	Retrospective, observational, monocentric study	2009–2014	IB1 > 2 cm^a^ IB^a^	9
Tesfai et al.^31^	Netherlands	Retrospective, observational, monocentric study	2006–2018	IB–IIA^a^	19
Testa et al.^32^	Italy	Retrospective, observational, multicentric study	2003–2008	IB1 > 2 cm^a^	6
Ungár et al.^33^	USA	Prospective, observational, monocentric study	1997–2002	IB1 > 2 cm^a^	10
Vercellino et al.^34^	Germany	Retrospective, observational, multicentric study	2004–2011	IB1 > 2 cm^a^ IB2^a^	6
Wethington et al.^35^	USA	Retrospective, observational, monocentric study	2001–2011	IB1 > 2 cm^a^	29
Zusterzeel et al.^36^	Netherlands	Retrospective, observational, monocentric study	2009–2018	IB2	18

^a^FIGO stage 2009

#### *FIGO* International Federation of Gynecology and Obstretics

The quality of all studies was assessed using the Newcastle–Ottawa scale^10^ (see the electronic supplementary material). Overall, the publication years ranged from 1991 to 2019. 691 patients who underwent FST for ECC ≥ 2 cm were enrolled in this systematic review. The follow-up period ranged from 21 to 95 months on average.

### Outcomes

A total of 691 patients were included in the review. Fourteen of the 26 selected studies presented data on surgical FST, while the remaining 12 studies reported on FST with the use of NACT. We summarized data from FSTs with upfront surgery and FSTs with an NACT approach.

### Surgical Fertility-Sparing Treatment (FST) Outcomes

Cao et al.^11^ performed a retrospective comparison between vaginal and abdominal trachelectomy in ECC. Data on ECC ≥ 2 cm treated with ART or VRT subanalysis were reported for a total of 48 patients (24 ART vs. 24 VRT). In a mean follow-up period of 20 months, no recurrence occurred in the ART group and 5 in the VRT group (RR 21.3% for VRT).

Deng et al.^13^ reported a 3-year DFS and OS rate of 95.2% and 97.6%, respectively, in 45 patients treated with ART.

Guo et al.^14^ investigated the oncological safety of ART compared with radical hysterectomy. In a subanalysis of this trial, 71 patients with ECC 2–4 cm underwent ART, with three recurrences and two deaths (RR 5.6%).

In 2010, Kim et al.^15^ treated eight patients with LRT for a 2–4 cm ECC, and reported a case of recurrence and subsequent death after 12 months (RR 12.5%).

In 2011, Li et al.^17^ published data on 14 ECCs of 2–4 cm treated with ART, with no recurrence in 22.8 months of mean follow-up.

In 2019, Li et al.^18^ expanded the case series with 132 patients and a 56-month follow-up period, during which they reported 11 recurrences and 5 deaths (RR 8.3%).

Litner et al.^19^ reported on 31 patients with ECC ≥ 2 cm treated with ART plus pelvic lymphadenectomy (PLND), 14 of whom had tumors ≥ 4 cm. Histotypes considered included grassy cell and anaplastic. These authors reported a median follow-up period of 90 months, with four recurrences and two deaths (RR 12.9%).

In 2020, Okugawa et al.^23^ reported on their retrospective experience of FST of ECC, comparing outcomes between patients with tumor sizes < 2 cm and ≥ 2 cm. Seventy-seven patients with ECCs ≥ 2 cm treated with ART had only two episodes of recurrence and one death from disease (RR 2.6%).

Pahisa et al.^24^ described their experience with VRT in ECC. In their series, four patients had tumors ≥ 2cm in size. Only one recurrence occurred (RR 25%), but 50% of the procedures were aborted and converted to radical hysterectomy because of intraoperative complications.

Park et al.^25^ treated 29 ECCs ≥ 2 cm with LRT and reported nine recurrences and one death (RR 31%).

In 2011, Plante et al.^26^ published a series of 125 VRTs preceded by laparoscopic PLND, of which 14 were patients with tumors ≥ 2 cm. Six cases of recurrence and two deaths were observed in this subanalysis (RR 42.9%).

A 2013 paper by Testa et al.^32^ described six cases of RT for ECCs > 2 cm—five squamous cell carcinomas and one adenocarcinoma with no episode of recurrence.

In a stratification of the data shown by Ungar et al.^33^ in 2005, data were obtained for 10 patients with tumors ≥ 2 cm undergoing ART + PLND who had no recurrence event in 47 months of median follow-up.

Finally, Wethington et al.^35^ published a case series of 29 patients with ECC ≥ 2 cm treated with both abdominal and laparoscopic trachelectomy as well as robotic trachelectomy. One case that underwent RRT showed the only recurrence in the series, a woman with a 3 cm tumor (RR 3.5%).

Overall, surgical FST techniques showed an RR of between 0 and 42.6%, with a median follow-up period of between 20 and 93 months. In contrast, the RR range was between 0 and 12.9% for ART alone. If we exclude papers that presented data related to special histotypes (other than squamous cell carcinoma, adenocarcinoma, and adenosquamous carcinoma), the RR was 0–8.3%. These results are summarized in Table 2.

**Table 2:** Surgical fertility-sparing treatment outcomes

References	No. of patients	Hystological subtype	Trachelectomy	Lymphadenectomy	Stromal infiltration > 50% (%)	Recurrence	Death	Mean follow-up	Recurrence rate (%)
Cao et al.^11^	48	SCC, ADK	24 ART/24 VRT	NR	4.17/4.17	0/5	NR	20	0/21.7
Deng et al.^13^	45	SCC, ADK	ART	PLND/PALND	0 (in recurrences)	2	1	61	4.4
Guo et al.^14^	71	SCC, ADK, ADS	ART	SLN	20.3	4	3	75.5	5.6
Kim et al.^15^	8	SCC, ADK	LRT	PLND/PALND	100	1	1	31	12.5
Li et al.^17^	14	SCC, ADK, ASK	ART	NR	NR	0	0	22.8	0
Li et al.^18^	132	SCC, ADK, ASK	ART	NR	13.8	11	5	56	8.3
Litner et al.^19^	31	SCC, ASK, ADK, Anaplastic, glassy cell	ART	PLND	NR	4	2	90	12.9
Okugawa et al.^23^	77	SCC, ASK, ADK	ART	SLN	NR	2	1	72	2.6
Pahisa et al.^24^	4	SCC, ADK	VRT	NR	NR	1	0	NR	25
Park et al.^25^	29	SCC, ASK, ADK	LRT	PLND	27.8	9	1	44	31
Plante et al.^26^	14	SCC, ADK	VRT	PLND	NR	6	2	93	42.9
Testa et al.^32^	6	SCC, ADK	ART	PLND	NR	0	0	29.6	0
Ungar et al.^33^	10	SCC, ADK, glassy cell	ART	PLND	NR	0	0	47	0
Wethington et al.^35^	29	SCC, ADK, ASK	22 ART/6 LRT/1 RRT	PLND	NR	1 (RRT)	0	44	3.5

*NR* not reported, *SCC* squamous cervical cancer, *ADK* cervical adenocarcinoma, *ASK* cervical adenosquamous carcinoma, *ADS* cervical adenosarcoma, *PLND* pelvic lymphadenectomy, *PALND* para-aortic lymphadenectomy, *SLN* sentinel lymph node, *RRT* robotic radical trachelectomy, *ART* abdominal radical trachelectomy, *VRT* vaginal radical trachelectomy, *LRT* laparoscopic radical trachelectomy

### Neoadjuvant Chemotherapy FST Outcomes

The paper by de Vincenzo et al.^12^ represents the earliest published observational study of NACT with three cycles of cisplatin and paclitaxel q21 in ECC ≥ 2 cm. Of the 13 eligible patients, 11 responded to treatment. Furthermore, this study has the highest rate of complete response reported to date (84.5%). One patient presented with stable disease and was treated with conventional standard radical surgery, however one patient died due to disease progression. A single recurrence was observed in the 11 treated patients, with a mean follow-up of 37 months (RR 15.4%).

Lanowska et al.^16^ published data on 18 patients treated with two or three cycles of paclitaxel + ifosfamide + cisplatin followed by radical vaginal trachelectomy (RVT). Complete response was obtained in 50% of the cases and only one recurrence was observed in a mean follow-up period of 23 months (RR 5.5%).

Lu et al.^20^ successfully treated six women with ECC ≥ 2 cm with two cycles of bleomycin + cisplatin + mitomycin q21 followed by LRT. At a 66-month median follow-up period, no recurrence was observed.

In 2011, Marchiole et al.^21^ presented a series of seven patients treated with three or four cycles of cisplatin + paclitaxel + ifosfamide, with a complete response in 57% of cases and an RVT on completion. No recurrences were reported. The same group reported an update of the case series in 2018,^22^ increasing the number of patients to 19, adding as an alternative to previous chemotherapy three to five cycles of cisplatin + paclitaxel + epirubicin, with CRR increased to 63%, and a completion surgery with laparoscopic-assisted vaginal trachelectomy (LARVT). Only two recurrences (RR 10.5%) were reported in a median follow-up period of 22–79 months.

Rendon et al.^27^ reported on the experience of 25 patients treated with different chemotherapy regimens combined with conization or radical trachelectomy, either by the open or laparoscopic approach. Complete response was observed in 44% of patients, with an RR of 12%.

In 2014, Robova et al.^28^ published data on dose-dense NACT (two different types based on histotypes) using an interval of 10–14 days. A combination of cisplatin (75 mg/m^2^) and ifosfamide (2 g/m^2^, maximal total dose 3 g) was used in squamous cell carcinoma, and cisplatin (75 mg/m^2^) plus doxorubicin (35 mg/m^2^) was used in all adenocarcinomas. After NACT, patients underwent ART or VRT. A complex response was preserved in 21.4% of cases, and the RR was 14.3%.

A subanalysis of the paper by Salihi et al.^29^ showed data from five patients with ECC ≥ 2 cm—one treated with three cycles of paclitaxel, ifosfamide and cisplatin, and four treated with three cycles of paclitaxel and carboplatin weekly. Of the five patients, three had a complete response, one treated with paclitaxel, ifosfamide and cisplatin had a partial response, and one patient had progression.

In 2016, Slama et al.^30^ reported data on nine patients treated with stroma. Ifosfamide 1.75 g/m^2^ in combination with cisplatin 75 mg/m^2^ was administered to patients with squamous cell cancers, while doxorubicin 35 mg/m^2^ or paclitaxel 75 mg/m^2^ in combination with cisplatin 75 mg/m^2^ was administered to patients with adenocarcinomas, both in ‘dose density’ (10–12 days) intervals. Two recurrences and one death were reported (RR 22.2%).

Tesfai et al.^31^ instead adopted an NACT regimen of six cycles of cisplatin and paclitaxel. Of the 19 patients treated, a complete response was observed in 26.3% of cases. All cases were completed with ART, and the RR was 15.8%.

Vercellino et al.^34^ published a paper in 2012 focusing on the role of lymphadenectomy as an assessment of the lymph node status of patients with ECC to be candidates for FST. Of the six patients with ECC ≥ 2 cm, cisplatin + paclitaxel ± ifosfamide, followed by VRT, resulted in a 50% CRR, with no recurrence during the median 30-month follow-up period.

Finally, Zusterzeel et al.^36^ proposed a six-cycle scheme of carboplatin + paclitaxel weekly in 18 patients with FIGO stage IB2, achieving a CRR of 38.8% and an RR of 22.2%. Overall, in a median follow-up period of between 22 and 79 months, the application of NACT schemes resulted in an RR of between 0 and 22.2%. In addition, wide variability in CRs was observed (21.4–84.5%).

Finally, in the reviewed papers, there was no agreement or standardization in the technique of deepening lymph node status, with surgeries ranging from sentinel lymph node (SLN) to systematic pelvic lymphadenectomy (PLND) and para-aortic lymphadenectomy (PALND). These results are summarized in Table 3.

**Table 3:** Neoadjuvant chemotherapy fertility-sparing treatment outcomes

References	No. of patients	Hystological subtype	Lymph node assesment	Chemotherapy	Surgery	Complete response (%)	Stromal Infiltration > 50% (%)	Recurrence	Death	Mean follow-up	Recurrence rate (%)
De Vincenzo et al.^12^	13	SCC, ADK	PLND/SLN	3 cisplatin + paclitaxel q21	Cold-knife conization	84.5	NR	2	1	37	15.4
Lanowska et al.^16^	18	SCC, ADK, ASK	PLND/SLN	2–3 cisplatin + paclitaxel + ifosfamide	VRT	50	NR	1	0	23	5.5
Lu et al.^20^	6	SCC	SLN	2 bleomycin + cisplatin + mitomycin q21	LRT	NR	NR	0	0	66	0
Marchiole et al.^21^	7	SCC, ADK	PLND	3–4 cisplatin + paclitaxel + ifosfamide	VRT	57	NR	0	0	22	0
Marchiole et al.^22^	19	SCC, ADK	PLND/SLN	3–5 TIP (cisplatin + paclitaxel+ ifosfamide) 3–5 TEP (cisplatin + paclitaxel + epirubicin)	LARVT	63	NR	2	0	79	10.5
Rendon et al.^27^	25	SCC, ADK	PLND /SLN	3–6 range carboplatin/paclitaxel (32%), cisplatin/paclitaxel (28%), paclitaxel/ifosfamide/cisplatin (16%), paclitaxel/cisplatin/5-fluorouracil (12%), 5-fluorouracil/ifosfamide/cisplatin (8%), vincristine/bleomycin/cisplatin in one patient	(5) Cold-knife conization/(11) ART/(9) LRT	44	NR	3	0	62	12
Robova et al.^28^	28	SCC, ADK	PLND/SLN	3 cisplatin + ifosfamide (SCC); cisplatin + doxorubicin (ADK)	ART/VRT	21.4	NR	4	2	42	14.3
Salihi et al.^29^	5	SCC, ADK	PLND	Cisplatin + paclitaxel + ifosfamide/carboplatin + paclitaxel weekly	Cold-knife conization	64	NR	1	0	58	20
Slama et al.^30^	9	SCC, ADK	SLN	3 cisplatin+ ifosfamide (SCC); cisplatin + doxorubicin (ADK)	Needle conization/simple vaginal trachelectomy	NR	NR	2	1	23	22.2
Tesfai et al.^31^	19	SCC, ADK, ADK with clear cell	PLND	6 cisplatin + paclitaxel	ART	26.3	NR	3	1	50	15.8
Varcellino et al.^34^	6	SCC, ADK, ASK	PLND/PALND	1 cisplatin + paclitaxel (16.6%) 2–3 cisplatin + paclitaxel + ifosfamide (83.4%)	VRT	50	NR	0	0	30.6	0
Zusterzeel et al.^36^	18	SCC, ADK	PLND	6 cisplatin + paclitaxel	VRT	38.8	NR	4	0	49.7	22.2

*NR* not reported, *SCC* squamous cervical cancer, *ADK* cervical adenocarcinoma, *ASK* cervical adenosquamouscarcinoma, *PLND* pelvic lymphadenectomy, *PALND* para-aortic lymphadenectomy, *SLN* sentinel lymph node, *VRT* vaginal radical trachelectomy, *LRT* laparoscopic radical trachelectomy, *LARVT* laparoscopic-assisted vaginal trachelectomy, *ART* abdominal radical trachelectomy

## Discussion

Selection of the ideal patient to be a candidate for FST is still the main problem in the management of ECC; however, women with tumors < 2 cm in size, younger than 40 years of age and desirous of offspring find FST feasible.^3,4^ The problem becomes bigger when the tumor size exceeds 2 cm. In a scenario in which FST is not represented by a single technique but by a combination of different approaches, tumors ≥ 2 cm is an area in which there is less concordance in the literature and less standardization of techniques.^5^ The scientific community comprises groups with a surgical-first approach and groups using NACT. A lack of standardization even for these two approaches makes clinical practice extremely heterogeneous. This situation is also due to another factor, i.e. tumor size is one of the main risk factors for patients with ECC but it is certainly not the only risk factor. There are several criteria to be considered to select a patient for FST. First, the assessment of lymph node status; only patients with negative lymph nodes can be assessed for FST.^3,4^ This observation implies that lymph node negativity should be ascertained before or during the procedure to allow the surgeon to abandon FST and change it into conventional surgery (radical hysterectomy). The methods used by various teams to establish lymph node negativity vary from imaging techniques alone to systematic pelvic and lumbar-aortic lymphadenectomy. It should be mentioned that tumors larger than 2 cm themselves represent a high-risk category for lymph node spread and therefore assessment of lymph node negativity could play a crucial role in patient selection. In the group of upfront surgery papers, with the exception of Cao et al.^11^, Li et al.^17^, Li et al.^18^, and Pahisa et al.^24^, whose papers do not report these data, all other papers used lymphadenectomy as a method of intraoperative lymph node assessment. The degrees of extension of the technique range from SLN to systematic pelvic and lumbar-aortic lymphadenectomy. However, this strategy has some limitations. The use of frozen section does not permit the characterization of micrometastases, which themselves represent an independent risk factor for recurrence.^37,38^ On the other hand, dividing the approach into two steps—first performing lymphadenectomy and then conservative surgery only on histologically proven lymph node-negative patients—could hypothetically increase the intraoperative difficulties due to tissue fibrosis, hypothetically increasing the morbidity of the techniques and their risk of abandonment.^39,40^ Unfortunately, in this situation of impasse, the approach to be used is completely left to the choice of the team. A future alternative could be represented by intraoperative amplification techniques to obtain information in frozen section about micrometastases,^41,42^ but to date does not represent a clinical standard. Vice versa, teams that prefer the use of NACT necessarily must resort to a prechemotherapy assessment of lymph node status. In these groups of patients, lymph node investigations range from SLN to lymphadenectomy. However, contrary to the purely surgical group, where lymphadenectomy may follow SLN, in these cases, the SLN alone may be useful to select patients but may not guarantee the patient the curative benefit related to lymphadenectomy.^43–45^ Moreover, patients undergoing NACT will complete their FST with surgical techniques that are as minimally invasive as possible. The goal of this approach is to minimize surgical morbidity and improve fertility outcomes, which were not the subject of this review. However, regarding this group of patients, the great heterogeneity in the therapy schemes applied should be noted, which led to extremely variable CRRs (21.4–84.5%). This additional variable represents a confounding factor in determining the best FST approach for patients with ECC ≥ 2 cm. Similarly, it has been almost impossible to extract from the published papers the rationale for the surgical approach chosen in combination with NACT. No paper expressed how much this should be influenced by the response of the tumor to chemotherapy. Furthermore, we have no evidence of different behavior between chemosensitive and partially sensitive tumors. While the first category may represent an ideal candidate for FST, the other may be potentially considered with a degree of aggressiveness that exposes the patient to a higher risk of recurrence. In addition, it should be considered that papers reporting the combination of NACT and ART are likely to sum up the comorbidities of both. This combination could invalidate fertility outcomes without a real advantage in terms of RR, substantially overlapping with fertility-sparing surgery alone. Another ideological problem of patients’ selection is related to histological characteristics, primarily the size. There is no agreement in the reported studies of which is the method of choice for measuring tumor size (clinical evaluation, MRI,^46^ or the performance of conization before FST), and most of the papers in the literature do not report these data. Furthermore, the prognostic weight exerted by tumor size remains controversial. Recent scientific evidence has shown that lateral tumor extension (HZTE), marked as the maximum tumor diameter, is subordinate to stromal infiltration.^47,48^ This concept has recently led to a revision of the FIGO stage IA and IB definitions in 2019.^49^ In contrast, there is evidence in the literature that excessive extensions > 4 cm have proven negative outcomes.^50^ Unfortunately, none of the studies reported data on stromal infiltration. This may represent patient selection bias, grouping together patients with different risk profiles. Similarly, histologic characterization provides other key risk factors to guide our choice. First is the histotype. In the literature, there is agreement that squamous histotypes and adenocarcinomas are candidates for FST, while histologies such as grassy cell, anaplastic, or clear cell should be considered as types at high risk of recurrence^6^ and should therefore be reserved for FST only in highly selected cases.^5^ Not surprisingly, the paper by Litner et al.,^19^ the only study among FST surgical approaches to have enrolled patients with special histotypes, is also the study with the highest RR for ART (12.9%). Adenosquamous tumors, which are considered an intermediate aggressiveness and therefore more suitable for FST, must be considered a borderline indication of FST.^51^ Other fundamental histological factors are represented by grading^52^ and depth of infiltration.^53^ These factors represent an incognita that often becomes known only after FST, and may condition the choice for adjuvant treatment^54^ or abandonment of FST, because punch biopsy alone may not represent the entire tumor.^55^ Finally, a key prognostic factor that is often missed in the decision-making process for FST is LVSI.^56,57^ In the case of positive LVSI, it is theoretically possible to hypothesize a greater benefit of NACT approaches because of the systemic bonification that they guarantee, and therefore greater control regarding distant recurrences, of which they can and do represent an independent risk factor.^58^ For the reasons stated, it might be routinely advised to perform conization before FST, regardless of the strategy to be practiced, with the advantage of obtaining this histological information and decreasing tumor size.^59^ The lack of standardization of the concepts of ‘close margin’ and ideal length of residual healthy cervical tissue is a final confounding factor, with the distance varying in the case series between 0.5 and 1 cm. Most of the proposed works do not report these characteristics, making a vain attempt to weigh the risks related to the technique adopted. In this regard, the radicality of the surgical approach adopted in FST seems to be crucial in the RR for patients with ECC ≥ 2 cm. In 2016, a review published in *Lancet Oncology*^5^ disputed the role of VRT and simple trachelectomy in patients with tumors ≥ 2 cm in size. Our results agree, showing that the VRT approach has the highest RR (Cao et al.^11^, 21.7%; Pahisa et al.^24^, 25%; Plante et al.^26^, 42.9%). These figures are very different from the results attributed to ART, where the RR is at a maximum of 5.6% (Guo et al.^14^). On the other hand, minimally invasive techniques deserve a separate discussion, where the RR of laparoscopic approaches has a peak of 31% in the study by Park et al.^25^ This funding aligns with the current scientific evidence regarding the best surgical approach for radical hysterectomy in tumors ≥ 2 cm.^8,60^ Even if heterogeneity of the adopted therapy schemes and the completion surgeries makes a direct comparison almost impossible, the group of works on NACT FST showed, on average, higher RR (5.5–22.2%) compared with ART (0–12.9%). Moreover, the surgical approach could condition the pattern of recurrence. Centro-pelvic recurrences should be ascribed to defects in surgical technique, whereas distant recurrences could be attributable to intrinsic tumor characteristics or poor patient selection.^61,62^

Our opinion is that the strength of the study lies in its systematic nature and rigor of the research, which has extracted all the literature data on patients with ECC ≥ 2 cm. Similarly, this represents the main limitation of our paper, which aims to summarize data from extremely heterogeneous approaches that well reflect current clinical practice. In addition, this review represents a partial view of the problem of fertility preservation, focused mainly on oncological outcomes. Our research group is currently conducting a similar review focused on fertility outcomes (Prospero registration number CRD42022329253). However, it seems clear that greater standardization in the selection of patients is necessary, and the identification of different risk classes even within a pattern of patients, i.e. those with ECC ≥ 2 cm, is already considered at the extreme limits of acceptability of FST. The higher the patient's inherent risk, the more attention should be paid by the clinician to the contextualization of the proposed clinical pathway. Therefore, it would be desirable to design clinical trials that prospectively minimize the bias related to tumor characteristics and not to the proposed FST.

## Conclusion

Our paper focused on the significant heterogeneity present in the clinical management of FST of ECC ≥ 2 cm. Nevertheless, from an oncological point of view, approaches limited to the minimally invasive or vaginal techniques seem to show the highest RR. Vice versa, the lack of standardization of NACT schemes and the wealth of confounders to be attributed to the histological features of the tumor make it difficult, if not impossible, to set a standard of treatment. Further randomized clinical trials with clear patient selection criteria will be necessary to clarify the existing doubts.

## Supplementary Information

Below is the link to the electronic supplementary material.

Supplementary file 1 (DOCX 16 kb)
