# Supplementary material for: Fertility-Sparing Treatment for Early-Stage Cervical Cancer ≥ 2 cm: A Problem with a Thousand Nuances—A Systematic Review of Oncological Outcomes
Source: Ann Surg Oncol. 2022 Sep 5;29(13):8346–58. doi: 10.1245/s10434-022-12436-w (PMC9640451; doi:10.1245/s10434-022-12436-w)
Supplement: Supplementary file 1 — (DOCX 16 kb) [file 10434_2022_12436_MOESM1_ESM.docx]

**Supplementaries. Newcastle–Ottawa scale**

| Single Arm Studies | | | | | | |
| --- | --- | --- | --- | --- | --- | --- |
| **Name** | **Country** | **Study design** | **Selection** | **Comparability** | **Outcome** | **Tot** |
| **Cao D.**  **2013** | China | Prospective  Case-control  Multicentric  Study | 3 | 2 | 1 | 6 |
| **De Vincenzo 2021** | Italy | Retrospective  Observational  Monocentric  Study | 3 | 0 | 3 | 6 |
| **Deng X.**  **2017** | China | Retrospective  Observational  Monocentric  Study | 3 | 0 | 3 | 6 |
| **Guo J.**  **2019** | China | Retrospective  Observational  Monocentric  Study | 3 | 0 | 3 | 6 |
| **Kim J.**  **2010** | Korea | Retrospective  Observational  Monocentric  Study | 3 | 0 | 3 | 6 |
| **Lanowska M. 2014** | Germany | Retrospective  Observational  Monocentric  Study | 3 | 0 | 2 | 5 |
| **Li J.**  **2011** | China | Retrospective  Observational  Monocentric  Study | 3 | 0 | 3 | 6 |
| **Li X.**  **2019** | China | Retrospective  Observational  Monocentric  Study | 3 | 0 | 2 | 5 |
| **Lintner B.**  **2013** | Hungary  UK  USA | Retrospective  Observational  Multicentric  study | 3 | 0 | 3 | 6 |
| **Lu Q.**  **2014** | China | Retrospective  Observational  Multicentric  study | 3 | 0 | 2 | 5 |
| **Marchiole P. 2011** | France | Retrospective  Observational  Monocentric  Study | 3 | 0 | 3 | 6 |
| **Marchiole P. 2018** | France | Retrospective  Observational Monocentric study | 3 | 0 | 2 | 5 |
| **Okugawa K. 2020** | Japan | Retrospective  Observational  Monocentric  Study | 3 | 0 | 3 | 6 |
| **Pahisa J.**  **2008** | Spain | Retrospective  Observational  Monocentric  Study | 2 | 0 | 2 | 4 |
| **Park J.**  **2014** | Korea | Retrospective  Observational  Multicentric  Study | 3 | 0 | 3 | 6 |
| **Plante M.**  **2008** | Canada | Prospective Observational  Monocentric  Study | 3 | 0 | 3 | 6 |
| **Rendón G. 2021** | Colombia | Retrospective  Observational  Monocentric  Study | 3 | 0 | 3 | 6 |
| **Robova H. 2014** | Czech Republic | Retrospective  Observational  Monocentric  study | 3 | 0 | 2 | 5 |
| **Salihi R.**  **2015** | Belgium | Retrospective  Observational  Monocentric  Study | 3 | 0 | 3 | 6 |
| **Slama J.**  **2016** | Czech Republi | Retrospective  Observational  Monocentric  study | 3 | 0 | 2 | 5 |
| **Tesfai F.**  **2020** | Netherlands | Retrospective  Observational  Monocentric  study | 3 | 0 | 3 | 6 |
| **Testa R.**  **2013** | USA | Retrospective  Observational  Multicentric  Study | 3 | 0 | 2 | 5 |
| **Ungár L.**  **2005** | USA | Prospective Observational  Monocentric  Study | 3 | 0 | 2 | 5 |
| **Vercellino G.**  **2012** | Germany | Retrospective  Observational  Multicentric  Study | 3 | 0 | 2 | 5 |
| **Wethington S. 2013** | USA | Retrospective  Observational  Monocentric  Study | 3 | 0 | 2 | 5 |
| **Zusterzeel P. 2020** | Netherlands | Retrospective  Observational  Monocentric  Study | 3 | 0 | 3 | 6 |
